# Elevated Neutrophil-to-Lymphocyte Ratio Predicts Early Aggravation of Organ Dysfunction in Septic Patients in the Emergency Department

**DOI:** 10.1097/CCE.0000000000001511

**Published:** 2026-11-10

**Authors:** Thomas Lafon, Anaëlle Nardot-Suchaud, Julien Vaidie, Thomas Daix, Guillaume Monneret

**Affiliations:** 1 Emergency Department, Limoges University Hospital Center, Limoges, France.; 2 Department of Clinical Investigation Center, Inserm CIC 1435, Limoges University Hospital Center, Limoges, France.; 3 Department of Unité Mixe Recherche, Inserm UMR 1092, University of Limoges, Limoges, France.; 4 Medical-Surgical Intensive Care Unit, Limoges University Hospital Center, Limoges, France.; 5 Immunology Laboratory, Hospices Civils de Lyon, Hôpital E. Herriot, Lyon France.; 6 Department of Immunology of Research Laboratory in Pathophysiology of Injury Induced Immunosuppression, Université de Lyon, EA 7426 “Pathophysiology of Injury-Induced Immunosuppression,” Université Claude Bernard Lyon 1, Lyon, France.

**Keywords:** biomarkers, clinical deterioration, emergency service, hospital, multiple organ failure, Sepsis

## Abstract

**OBJECTIVES:**

To investigate whether neutrophil-to-lymphocyte ratio (NLR), a simple marker of immune dysregulation, is associated with early organ dysfunction progression in emergency department (ED) patients with sepsis.

**DESIGN:**

Exploratory secondary analysis of observational clustering cohort.

**SETTING:**

Single-center ED.

**PATIENTS/SUBJECTS:**

Among 999 patients with adjudicated infection, 364 fulfilled Sepsis-3 criteria for sepsis or septic shock and had available Sequential Organ Failure Assessment (SOFA) scores at admission and 24 hours.

**INTERVENTIONS:**

None.

**MEASUREMENTS AND MAIN RESULTS:**

Organ dysfunction trajectory was defined according to the evolution in SOFA score during the first 24 hours (ΔSOFA) and categorized as improving (ΔSOFA ≤ −2), stable (ΔSOFA −1 to +1), or worsening (ΔSOFA ≥ +2). Of the 364 patients, 135 (37%) were classified as improving, 187 (51%) as stable, and 42 (12%) as worsening. NLR differed significantly across trajectory groups, with median values of 12 (7–21), 12 (7–22), and 23 (10–37), respectively (*p* = 0.015). In multivariable logistic regression adjusted for age, lactate concentration, and baseline SOFA score, NLR remained independently associated with worsening organ dysfunction trajectory (odds ratio [OR] 5.55, 95% CI, 1.86–18.18; *p* = 0.003). Patients with worsening trajectories more frequently required ICU admission (26% vs. 5% vs. 4%; *p* < 0.001) and experienced higher 28-day mortality (36% vs 17% vs 17%; *p* = 0.013).

**CONCLUSIONS:**

NLR was independently associated with early worsening of organ dysfunction in ED patients with sepsis. Beyond its prognostic value, NLR may provide complementary information to conventional severity assessment by reflecting immune dysregulation.

**KEY POINTS**

**Question**: Does the neutrophil-to-lymphocyte ratio (NLR) predict early organ dysfunction progression in emergency department patients with sepsis?

**Findings**: Higher NLR at admission was independently associated with worsening organ dysfunction within 24 hours (odds ratio 5.55). Patients with worsening dysfunction more often required ICU admission (*p* < 0.001) and had higher 28-day mortality (*p* = 0.013).

**Meaning**: NLR may serve as a simple, prognostic marker for early sepsis severity, complementing traditional assessments by reflecting immune dysregulation.

Sepsis is increasingly recognized as a heterogeneous syndrome resulting from complex interactions between pathogen characteristics, host susceptibility, and the biological response to infection. Current severity assessment relies on the Sequential Organ Failure Assessment (SOFA) score and has recently undergone substantial refinement through the development of SOFA-2 (1). Despite these advances, the newly proposed score still fails to incorporate immune parameters. Consequently, the relationship between early immune dysregulation and subsequent organ dysfunction trajectories remains poorly characterized.

Among parameters reflecting the host immune-inflammatory response, the neutrophil-to-lymphocyte ratio (NLR) is a simple but practical marker that integrates both innate (i.e., neutrophils) and adaptive (i.e., lymphocytes) immune activity. In addition, elevated NLR has recently been shown to strongly correlate with decreased monocytic HLA-DR (mHLA-DR) expression, currently considered the reference biomarker for monitoring immune function in septic patients (2). Furthermore, early low mHLA-DR expression has been implicated in clustering patterns associated with clinical deterioration in patients with community-acquired pneumonia in the emergency department (ED) (3).

Although elevated NLR has primarily been associated with mortality in sepsis, we therefore hypothesized in the present study that this bedside surrogate of host response may be also associated with very early progression of organ dysfunction, a relationship that remains largely underexplored in the ED setting. To test this hypothesis, we leveraged a recent study in which we identified distinct subphenotypes in the ED and demonstrated their association with early clinical deterioration (4).

## METHODS

We performed an exploratory secondary analysis of a previously published retrospective observational cohort conducted in the ED and designed to identify subphenotypes associated with early clinical deterioration (4). This was an ancillary analysis of a previous retrospective observational cohort (4) that had received approval from an ethics committee (Number 38-2025-06 on July 28, 2025), who waived the need for informed consent due to the retrospective design. Thus, no institutional review board review was necessary for this new analysis as every ancillary analyses fall under the scope of the original ethics committee approval. The analysis was performed in accordance with the ethical standards of the responsible ethics committee and with the Helsinki Declaration of 1975. In this study, all infections were adjudicated by a multidisciplinary expert committee using clinical, microbiologic, radiologic, and follow-up data. For the present analysis, we restricted the population to patients fulfilling Sepsis-3 criteria for sepsis or septic shock. Early organ dysfunction trajectory was assessed using the variation in SOFA score between ED admission (H0) and a follow-up assessment within a predefined 24-hour window (24 ± 6 hr). ΔSOFA was calculated as the difference between the follow-up and baseline SOFA H0. Patients were categorized as improving (ΔSOFA ≤ −2), stable (ΔSOFA between −1 and +1), or worsening (ΔSOFA ≥ +2). The primary outcome was worsening organ dysfunction, defined as a SOFA increase of at least two points within 24 hours. NLR was calculated from the first routine neutrophil and lymphocyte counts obtained at ED admission.

Continuous variables were assessed for normality and are presented as median (Q1–Q3), and categorical variables as counts (percentages). Comparison were performed using Kruskal–Wallis tests and categorical variables using χ^2^ or Fisher exact tests, as appropriate. Correlations between NLR and conventional severity markers were assessed using Spearman coefficients. Because of its right-skewed distribution, NLR was log-transformed before regression analyses. Multivariable logistic regression was performed to evaluate the independent association between admission NLR and worsening organ dysfunction after adjustment for age, admission lactate concentration, and baseline SOFA score. Statistical analyses were performed using GraphPad Prism, Version 8 (GraphPad Software, San Diego, CA).

## RESULTS

Among 999 patients included in the original cohort, 364 fulfilled Sepsis-3 criteria (330 sepsis and 34 septic shock). According to early organ dysfunction trajectory, 135 patients (37%) were classified as improving, 187 (51%) as stable and 42 (12%) as worsening. Age and Charlson comorbidity index were similar across groups. In contrast, patients with worsening trajectories more frequently exhibited lower mean arterial pressure (84 [67–105] vs. 98 [83–115] and 93 [79–105] mm Hg, *p* = 0.005), and higher lactate concentrations (2.6 [1.5–4.1] vs. 1.7 [1.2–2.6] and 1.7 [1.2–2.5] mmol/L, *p* = 0.013). Baseline SOFA scores differed modestly across groups (median 3 [2–5], 3 [2–4], and 3 [2–6], respectively; *p* = 0.026) (**Table 1**). Among leukocyte subsets, lymphocyte counts were lower in patients with worsening (0.47 [0.32–0.95] G/L) compared with improving (0.78 [0.45–1.27] G/L) and stable patients (0.73 [0.48–1.08] G/L; *p* = 0.031), whereas neutrophil counts did not significantly differ between groups (*p* = 0.275) (Table 1).

**TABLE 1.** Baseline Characteristics and Clinical Outcomes According to Organ Dysfunction Evolution Assessed by Variation in Sequential Organ Failure Assessment Score During the First 24 Hours

Characteristics	Improving, *n* = 135	Stable, *n* = 187	Worsening, *n* = 42	*p*
Age (yr)	77 (64–87)	78 (69–85)	75 (62–84)	0.562
Male	77 (57%)	111 (59%)	25 (59%)	0.907
Charlson comorbidities index	5 (4–8)	6 (4–8)	6 (4–7)	0.588
Cancer/hemopathy	51 (37%)	55 (29%)	12 (29%)	0.243
Site of infection				0.034
Pulmonary	90 (67%)	100 (53%)	23 (54%)	
Abdominal	17 (49%)	19 (10%)	8 (19%)	
Urinary	12 (9%)	40 (21%)	5 (12%)	
Others	16 (12%)	28 (15%)	5 (12%)	
Documented infection	110 (81%)	150 (80%)	34 (81%)	0.959
Septic severity				
SOFA score H0	3 (2 to 5)	3 (2 to 4)	3 (2 to 6)	0.026
SOFA score at H24 ± 6	0 (0 to 1)	3 (2 to 4)	4 (2 to 6)	< 0.001
Δ SOFA score between H0 and H24 ± 6	–2 (–3 to –2)	0 (–1 to 0)	2 (2 to 4)	< 0.001
Lactates (mmol/L)	1.7 (1.2 to 2.6)	1.7 (1.2 to 2.5)	2.6 (1.5 to 4.1)	0.013
Mean arterial pressure (mm Hg)	98 (83 to 115)	93 (79 to 105)	84 (67 to 105)	0.005
Biology				
C-reactive protein (mg/L)	101 (40 to 194)	104 (45 to 197)	202 (60 to 344)	0.010
Procalcitonin (ng/mL)	0.85 (0.16 to 2.77)	0.84 (0.18 to 4.65)	2.45 (0.42 to 27.2)	0.095
Neutrophil-to-lymphocyte ratio	12 (7 to 21)	12 (7 to 22)	23 (10 to 37)	0.015
Neutrophils (G/L)^a^	9.8 (6.3 to 13.9)	9.3 (5.8 to 12.9)	10.3 (6.4 to 16.9)	0.275
Lymphocytes (G/L)^a^	0.78 (0.45 to 1.27)	0.73 (0.48 to 1.08)	0.47 (0.32 to 0.95)	0.031
Outcome				
ICU admission	7 (5%)	7 (4%)	11 (26%)	< 0.001
D-28 mortality	23 (17%)	31 (17%)	15 (36%)	0.013
Hospital length of stay	10 (5 to 15)	9 (5 to 15)	9 (4 to 12)	0.668

SOFA = Sequential Organ Failure Assessment.

a Comparisons were performed using the Kruskal–Wallis test.

Patients were categorized as improving (ΔSOFA ≤ −2), stable (ΔSOFA −1 to +1), or worsening (ΔSOFA ≥ +2). Results are expressed as median (Q1–Q3) or as *n* (%).

NLR differed significantly according to organ dysfunction evolution, with median values of 12 [7–21] in improving patients, 12 [7–22] in stable patients and 23 [10–37] in worsening patients (*p* = 0.015) (**Fig. 1*A***). The proportion of patients experiencing worsening organ dysfunction increased progressively across admission NLR quartiles (**Supplementary Fig.** https://links.lww.com/CCX/B702). Importantly, NLR demonstrated only weak correlations with baseline SOFA score (*r* = 0.04), lactate concentrations (*r* = 0.19), C-reactive protein (CRP) (*r* = 0.23) and procalcitonin (PCT) (*r* = 0.27), suggesting that it captures biological information distinct from conventional markers of severity (**Fig. 1*B***). In multivariable analysis, NLR remained independently associated with worsening organ dysfunction trajectory (OR 5.55; 95% CI, 1.86–18.18; *p* = 0.003), whereas SOFA score was not (OR 1.04; 95% CI, 0.81–1.32; *p* = 0.755) (**Fig. 1*C***). Patients exhibiting worsening trajectories more frequently required ICU admission (26% vs. 5% and 4%, *p* < 0.001) and experienced higher 28-day mortality (36% vs. 17% and 17%, *p* = 0.013) (Table 1). Admission NLR was higher in patients subsequently admitted to the ICU than in those not admitted to the ICU (22 [8–35] vs. 12 [7–23], *p* = 0.020), whereas NLR did not differ between patients who died within 28 days and survivors (13 [6–29] vs. 13 [7–24], *p* = 0.739).

**Figure 1.**
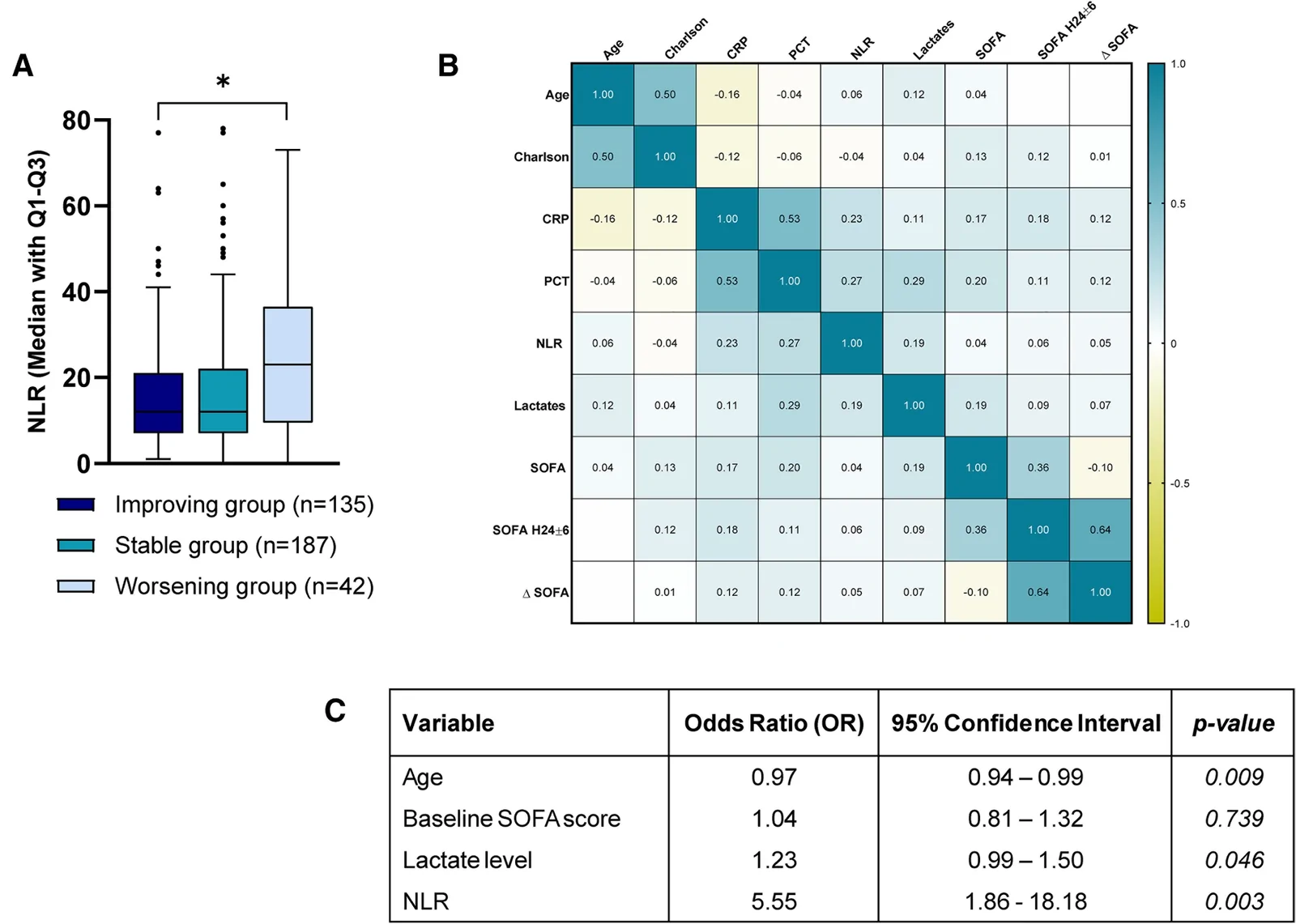
Association between admission neutrophil-to-lymphocyte ratio (NLR) and early organ dysfunction progression in sepsis. **A**, Distribution of NLR according to organ dysfunction evolution. *Boxes* represent median with Q1–Q3. Comparisons were performed using the Kruskal–Wallis test. **B**, Spearman correlation matrix. **C**, Multivariable logistic regression analysis evaluating factors independently associated with worsening organ dysfunction. Odds ratios (OR) are shown with 95% CIs. NLR was log-transformed before inclusion in the model. CRP = C-reactive protein, PCT = procalcitonin, SOFA = Sequential Organ Failure Assessment. *p value < 0.05.

## DISCUSSION

These findings suggest that NLR may capture a dimension of host-response dysregulation that is not reflected by conventional severity assessment using the SOFA score. Previous studies have primarily evaluated NLR as a predictor of mortality. For example, Suba et al (5) recently reported that early changes in NLR outperform baseline inflammatory markers in predicting 28-day mortality. Our findings extend this literature by showing that an elevated NLR predicts early worsening of organ dysfunction. This observation is clinically relevant because it may facilitate the early identification of patients at highest risk of deterioration, thereby supporting risk stratification and the timely implementation of enhanced care pathways.

Most importantly, NLR demonstrated only weak correlations with baseline SOFA score, lactate concentration, CRP, and PCT, yet remained independently associated with unfavorable outcomes in the multivariable model, whereas the other inflammatory parameters did not. This observation supports the concept that very early biological signals available at ED admission may already reflect divergent pathophysiological trajectories despite relatively similar initial clinical presentations. This interpretation is consistent with our previous subphenotype analysis, in which immune-related variables contributed to the identification of clusters associated with early deterioration despite limited differences in baseline severity (3, 4). In other words, these findings suggest that specific dimensions of the host response, not fully captured by SOFA or conventional inflammatory biomarkers such as CRP, may anticipate the progression of organ dysfunction at a stage when clinical severity appears comparable. One explanation may lie in the fact that NLR integrates two major components of the host response in sepsis: activation of innate immunity (reflected by neutrophil release from the bone marrow) and deactivation of adaptive immunity (reflected by lymphopenia), resulting in the concomitant expression of hyperinflammation and immunosuppression. This interpretation is supported by our recent demonstration of a strong relationship between elevated NLR and low mHLA-DR in patients with septic shock (2). Thus, beyond reflecting disease severity, NLR may provide prognostic information that is complementary to conventional biomarkers and clinical parameters (6).

Finally, focusing on ΔSOFA rather than mortality may represent a more physiologically relevant approach to investigating the clinical utility of host-response biomarkers. Interestingly, admission NLR was associated with subsequent ICU admission but not with 28-day mortality, further supporting the relevance of early organ dysfunction trajectories as an outcome of host-response dysregulation. Mortality is influenced by numerous factors occurring after ED admission, including therapeutic interventions, treatment limitations, and comorbidities. In contrast, early trajectories of organ dysfunction more directly reflect the immediate interaction between infection and host response.

Several limitations should be acknowledged: 1) this study represents a secondary analysis, 2) NLR was measured only at ED admission without longitudinal assessment, 3) the number of patients exhibiting worsening organ dysfunction was relatively limited, which precludes concluding on the association with mortality. Larger studies would be required on the subject, 4) although ΔSOFA is a clinically relevant endpoint, it remains partly influenced by therapeutic interventions during the first hours of management in ED, 5) specific information regarding preexisting immunosuppression was not systematically available, although underlying malignancy and hematologic malignancy were reported separately. Given these limitations, these findings should be considered exploratory and require external validation.

## CONCLUSIONS

In conclusion, NLR may represent a simple but highly practical marker of host-response dysregulation associated with divergent early trajectories of organ dysfunction. These findings support further investigation in the ED, which should be extended and validated in larger, multicentric cohorts. Upon confirmation, NLR assessment could complement conventional evaluations of organ dysfunction in sepsis.

## Supplementary Material
