# Supplementary material for: Elevated Neutrophil-to-Lymphocyte Ratio Predicts Early Aggravation of Organ Dysfunction in Septic Patients in the Emergency Department
Source: Crit Care Explor. 2026 Nov 10;8(11):e1511. doi: 10.1097/CCE.0000000000001511 (PMC13750867; doi:10.1097/CCE.0000000000001511)
Supplement: Supplementary file 1 [file cc9-8-e1511-s001.pdf]

Elevated Neutrophil-to-lymphocyte ratio predicts early aggravation of organ dysfunction in septic patients in the Emergency Department

Supplementary Figure - Proportion of patients experiencing worsening organ dysfunction according to neutrophil-to-lymphocyte ratio (NLR) quartiles.

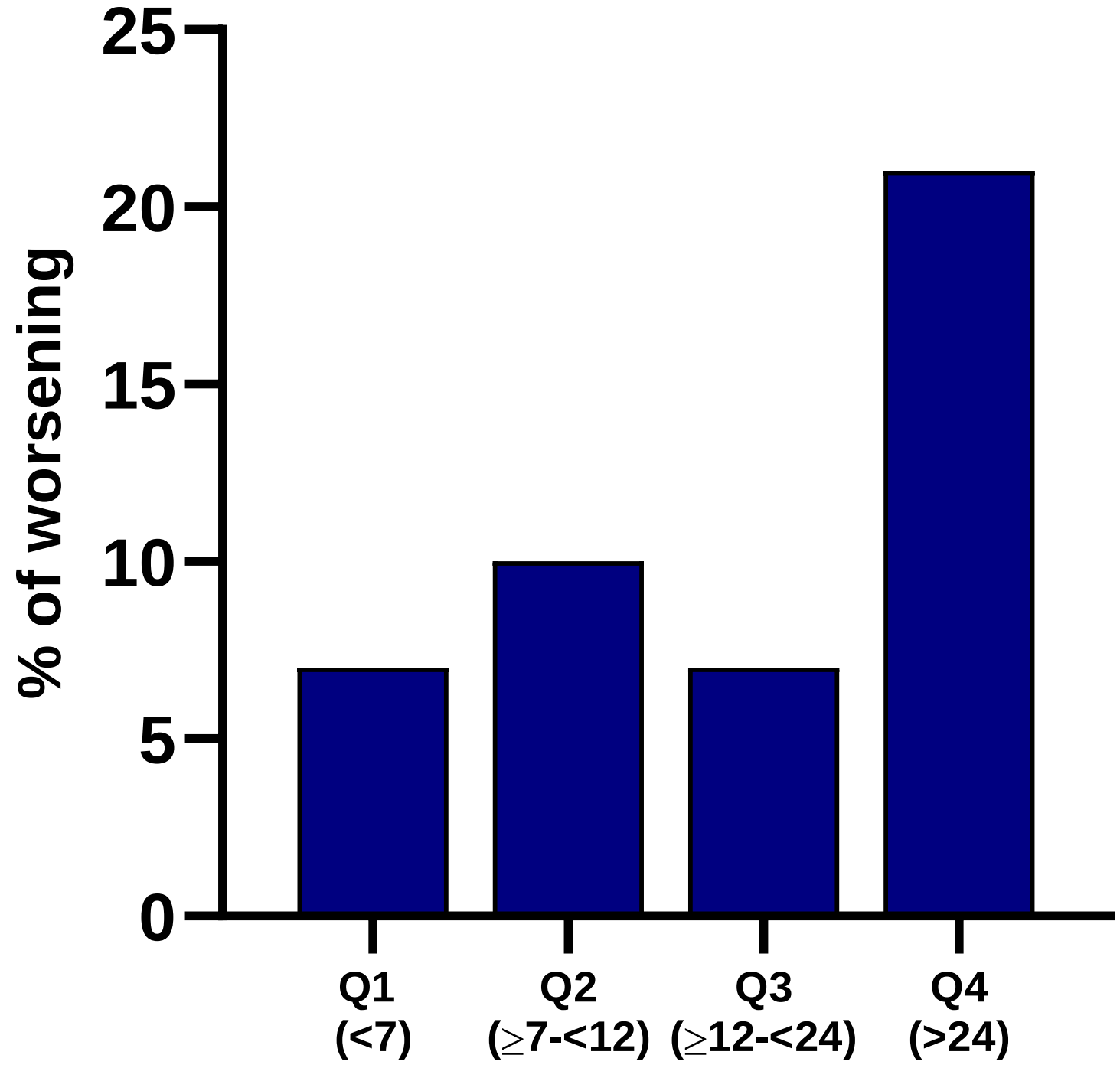

Worsening was assessed between admission in the Emergency Department (H0) and a follow-up assessment within a predefined 24-hour window.
